# Furfural Production from d-Xylose and Xylan by Using Stable Nafion NR50 and NaCl in a Microwave-Assisted Biphasic Reaction

**DOI:** 10.3390/molecules21081102

**Published:** 2016-08-22

**Authors:** Sarah Le Guenic, David Gergela, Claire Ceballos, Frederic Delbecq, Christophe Len

**Affiliations:** 1 Université de Technologie de Compiègne (UTC), CS 60319, 60203 Compiègne Cedex, France; sarah.le-guenic@utc.fr; 2 Department of Chemistry, Faculty of Technology, 760 01 Zlin, Czech Republic; David.Gergela@seznam.cz; 3 Ecole Supérieure de Chimie Organique et Minérale (ESCOM), 1 rue du Réseau Jean-Marie Buckmaster, 60200 Compiègne, France; c.ceballos@escom.fr (C.C.); f.delbecq@escom.fr (F.D.)

**Keywords:** furfural, d-xylose, xylan, Nafion NR50, biphasic system, microwave-assisted dehydration

## Abstract

Pentose dehydration and direct transformation of xylan into furfural were performed in a water-cyclopentyl methyl ether (CPME) biphasic system under microwave irradiation. Heated up between 170 and 190 °C in the presence of Nafion NR50 and NaCl, d-xylose, l-arabinose and xylan gave furfural with maximum yields of 80%, 42% and 55%, respectively. The influence of temperature and reaction time on the reaction kinetics was discussed. This study was also completed by the survey of different reactant ratios, such as organic layer-water or catalyst-inorganic salt ratios. The exchange between proton and cation induced by an excess of NaCl was monitored, and a synergetic effect between the remaining protons and the released HCl was also discovered.

## 1. Introduction

Because of the depletion of fuel-based resources, there is an urgent need to find alternative feedstocks for the chemical industry. Lignocellulosic biomass, plant wastes for example, appears to be the best alternative to get access to various bio-based chemicals, such as furan derivatives. One of these top-value materials is furfural, largely produced from the dehydration of d-xylose, the main monomer of hemicellulose-rich biomass.

Furfural is a key compound for the preparation of many intermediates [1]. For example, furfuryl alcohol is a precursor of commercially available resins (namely polyfurfuryl alcohol or PFA) and can be formed from furfural by the reduction of its aldehyde function. Furfural is also useful for the preparation of organic solvents, such as tetrahydrofuran (THF), methyltetrahydrofuran (MeTHF) or the synthesis of bioactive compounds. The industrial production of furfural is realized by batch and continuous acid hydrolysis of lignocellulosic biomass [2] into d-xylose followed by its dehydration leading to furfural. Both acid hydrolysis and subsequent dehydration of lignocellulosic biomass are usually carried out in the same reactor, and that dehydration is recognized as the rate-limiting step. The reaction is usually carried out in a range of temperatures found between 170 and 185 °C under a conventional heating. Performed in water, these processes require strong mineral acids, such as HCl [3,4], H_2_SO_4_ [5] or H_3_PO_4_ [6], causing corrosion, safety and handling problems. Recovery of furfural requires distillation of the solvent and other treatments, such as neutralization, which lead to large waste streams and high-energy consumption. Besides, in a monophasic system, furfural yields and selectivity are generally limited by the formation of by-products, humin, a black insoluble carbonaceous solid, derived from the cross-polymerization between furfural and pentose intermediates, or from the condensation of furfural on itself.

Today, the use of easy-to-handle solid sulfonated polymers recognized for their strong Brønsted acidity, such as protonated Amberlyst [7,8,9,10,11] or Nafion [12], shows promising results in the field of green chemistry. Nafion NR50 is a perfluoroalkane sulfonic resin that could be an alternative to other types of heterogeneous catalysts, such as zeolites [13] or heteropolyacids [14]. According to the producer data, Nafion NR50 has a maximum operating temperature of 220 °C. Recently, Lam et al. demonstrated the potential of the solid Nafion 117 for the C5 sugar dehydration [15]. They reported the formation of furfural with a maximum yield of 60% at 150 °C in dimethylsulfoxide (DMSO). However, during the process, Nafion was also subjected to the deposition of a humin layer. This layer almost covered the entire active surface of the solid catalyst and limited the number of catalytic runs. Nafion NR50 resin was also reported as an efficient superacid catalyst in the presence of ionic liquids for the hydrolysis of cellobiose into d-glucose monomers [16]. To inhibit the formation of side products like humins, one of the most promising approaches is to add an organic co-solvent, such as cyclopentyl methyl ether (CPME), a low water-miscible eco-friendly solvent [17]. The use of an organic co-solvent allows the extraction of furfural from the aqueous phase into the organic phase and thus improves the furfural yield by inhibiting the generation of undesired products. With regards to the scale-up process, in order to save energy and avoid undesirable degradations, it is better to use extractive solvent with lower boiling points than that of solubilized furfural to easily recover the target compound. According to Weingarten et al. [18], a biphasic system does not alter the fundamental kinetics of its entire reaction compared to the classic monophasic system. Its only role is to be the storage for the hydrophobic species. Several studies have reported the use of MIBK, toluene, nitrotoluene or dichloromethane as efficient co-solvents [19,20,21,22] for the dehydration of d-xylose. In accordance with a recent work of our group on the dehydration of d-xylose with homogeneous catalysis [23], the present work focuses on the use of the Nafion NR50 as a heterogeneous catalyst in the presence of NaCl in a biphasic system under microwave irradiation in a single mode cavity.

## 2. Results and Discussion

### 2.1. Influence of the Temperature on Furfural Yield

**Figure 1:**
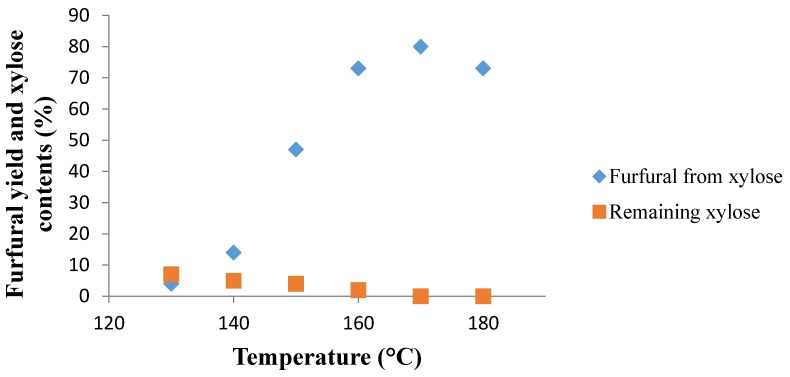
Effect of reaction temperature on furfural yield. Reaction conditions: Nafion NR50 (two pellets), NaCl (95 mg), d-xylose (1.0 mmol), water (1 mL), CPME (3 mL) and MW, 1 h.

Using the first results obtained by the group, the biphasic experiments were performed by employing two immiscible phases, water-CPME with a ratio 1:3 (*v*/*v*) [23]. d-xylose is not soluble in the organic phase, and CPME is quite stable at high temperature. Due to its low density, this extraction solvent was the upper phase. The studies of the dehydration of d-xylose were conducted for 1 h between 130 and 180 °C in the presence of NaCl (95 mg) and two Nafion NR50 pellets in a biphasic mixture (water-CPME, 1:3, *v*/*v*) under microwave irradiation (Figure 1). A maximum furfural yield of 80% was reached at 170 °C after one hour of reaction with a quantitative conversion of d-xylose, which is a competitive result by comparison with one of our most recent results of a 74% furfural yield obtained with FeCl_3_ as the catalyst in a biphasic system [23]. Between 130 and 160 °C, remaining d-xylose loading decreased slowly. It was noteworthy that Nafion NR50 pellets are subjected to a natural swelling in water at room temperature [24]. In a preliminary study, the same reaction was performed without the use of NaCl. However, Nafion NR50 pellets turned to black and exploded, and furfural yields were not reproducible. As a consequence, NaCl was necessary to keep the activity and the integrity of Nafion NR50 at 170 °C. The mechanism involved during the addition of NaCl will be described later in the publication. Besides, for comparison, the same reaction was carried out in a sealed tube filled with 4.0 mL of water-CPME (1:3, *v*/*v*) keeping unchanged the current substrate to catalyst ratio in the presence of 95 mg of NaCl. The dehydration was realized by means of a preheated oil bath with conventional heating and led to a furfural yield of only 55% at 150 °C for 2 h. Thereby, these results underline that the catalytic system Nafion NR50-NaCl could be an alternative to the common hydrochloric acid aqueous solution under microwave activation at 170 °C.

When the same procedure was applied to l-arabinose, a lower yield (42%) was obtained for 1 h at 170 °C, as has been observed in the literature [25]. It was reported that l-arabinose was dehydrating less rapidly than xylose at 170 °C [25,26]. This difference is due to the fact that d-xylose expressed lower activation energy for its dehydration compared to l-arabinose, generally superior to 112 kJ·mol^−1^. The structure of l-arabinose was more stable. The conversion of l-arabinose into furfural was also reported to be more temperature dependent and to require high temperatures (above 220 °C) to accelerate the process [27,28].

### 2.2. Effect of d-xylose Loading Variation

The influence of the initial d-xylose concentration was also studied in a mixture of water-CPME, 1:3, *v*/*v*, at 170 °C for 1 h under microwave irradiation (Figure S1, ESI). For a d-xylose concentration of 150 g·L^−1^ in the aqueous phase, the yield reached a value of 80%. Then, by keeping the optimized reaction conditions, the pentose loading was gradually increased until a singular decrease of the furfural yield was recorded. For a d-xylose concentration of 500 g·L^−1^, the furfural yield still reached a good value of 72%. However, if the concentration were increased to 600 g·L^−1^, the furfural yield dropped to the lower value of 62%. First, the high pentose concentration favored the contact between molecules and thus promoted condensation between pentose intermediates and furfural: larger amounts of a dark brown syrup insoluble in both layers were produced. Then, the large amounts of furfural produced from the high xylose concentration may also saturate the organic phase and make the furfural extraction no longer efficient. Finally, the viscosity of the reaction medium also increased with increasing substrate concentration and may have led to a non-uniform heating in the microwave reactor.

### 2.3. Influence of the Residence Time on the Furfural Production

**Figure 2:**
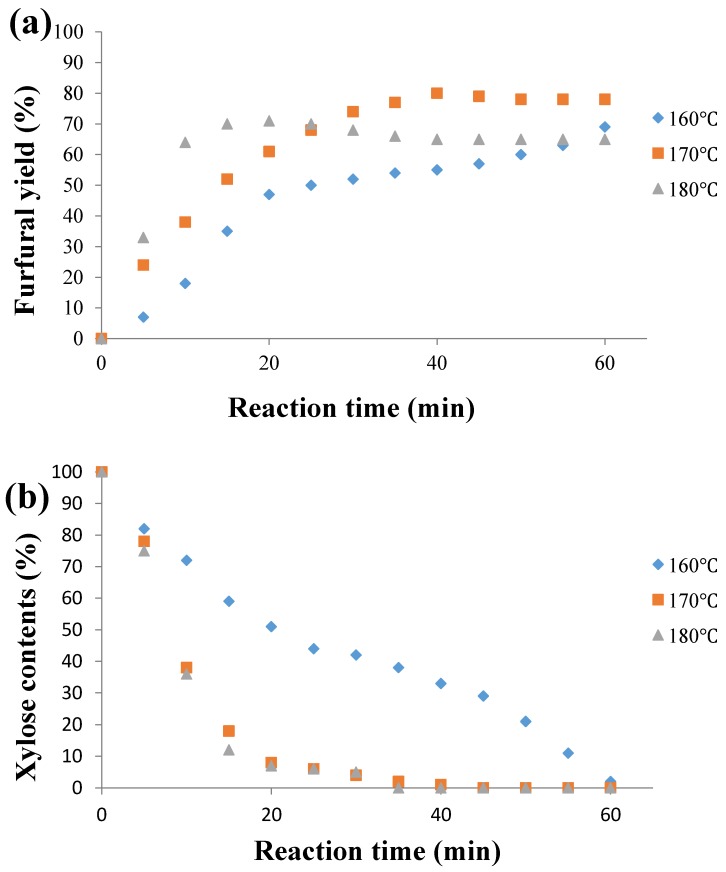
Effect of reaction time and temperature on (**a**) furfural yield and (**b**) xylose contents. Reaction conditions: Nafion NR50 (2 pellets), NaCl (95 mg), d-xylose (1.0 mmol), water (1 mL), CPME (3 mL) and MW.

After determining the best temperature condition for the dehydration of d-xylose using CPME as the extraction solvent, the effect of the reaction time was studied at 160 °C, 170 °C and 180 °C (Figure 2). Consequently, different time periods (from 5 to 60 min) were tested in order to determine the optimal conditions. The other parameters were unchanged using d-xylose (1.0 mmol), Nafion NR50 loading (two pellets), NaCl (95 mg) and a biphasic ratio water-CPME, 1:3, *v*/*v*. The trend of the d-xylose conversion curve did not change compared to those obtained from a typical monophasic batch operated in the same range of temperatures reported in Figure 2. After 25 min of fast consumption, only 5% of the d-xylose remained in the mixture. A maximum furfural yield of 80% was reached after 40 min of reaction. After this period, a slight decrease of the yield was observed. In addition, at 170 °C, the selectivity of the produced furfural never exceeded 80%, and only a small amount of humin was detected in the reaction vessel. In the opposite case, when the reaction was performed at 160 °C, the furfural formation was accelerated before 20 min, and the yield continued to increase slowly until it reached 69% after 60 min. At 180 °C, for a short period comprised between 10 and 20 min, furfural was produced with a high yield of 71%, but beyond this time, the furfural concentration started to decline due to its resinification, more effective at this temperature. Thus, the optimized reaction time and temperature were clearly defined. In order to decrease the reaction time, the temperature was fixed at 170 °C for 40 min under microwave irradiation. 

### 2.4. Effect of the Ratio Water-CPME on the Furfural Yield

**Figure 3:**
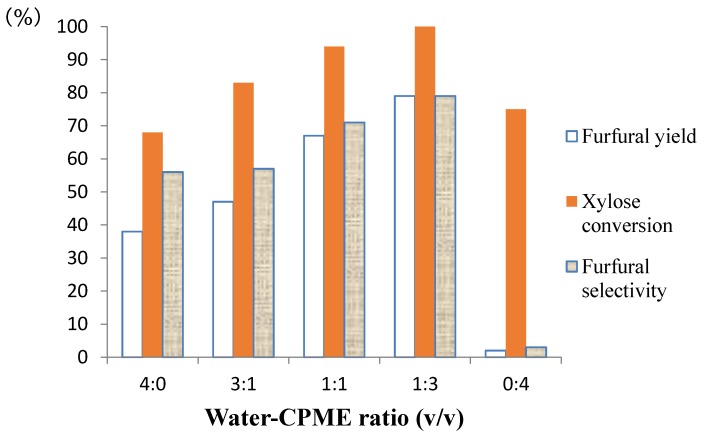
Effect of the water-CPME volumetric ratio on dehydration of d-xylose to furfural. Reaction conditions: Nafion NR50 (2 pellets), NaCl (95 mg), d-xylose (1.0 mmol), water-CPME, MW, 170 °C and 40 min.

In order to evaluate the effect of the ratio water-CPME on d-xylose conversion and furfural yield, five ratios of water-CPME (4:0, 3:1, 1:1, 1:3, 0:4; *v*/*v*) were studied (Figure 3). When the reaction was performed in pure water, as expected, the yield of the produced furfural never exceeded 38%, and the selectivity was moderate with a value of 57%. Besides, the conversion of d-xylose was not complete since the Nafion pellets deactivated during the process because of the deposition of a large amount of humins on the catalyst surface. By increasing the water-CPME ratio, the selectivity of furfural increased with values of 56%, 71% and 79% for water-CPME volumetric ratios of 3:1, 1:1 and 1:3, respectively. Furthermore, the coefficient partition found in the different water-CPME ratios above was also determined as follow. At 170 °C, 1.0 mmol of furfural was partitioned between the two phases in the presence of the catalytic system, and the value was calculated using this formula: Kp = [furfural]_water_/[furfural]_CPME_. According the HPLC measurement, except a slight decrease of furfural concentration in both layers, by increasing the volume of CPME, the coefficient remained almost constant until it reached a final value of 4.1 for the water-CPME (1:3, *v*/*v*) volume ratio. When the reaction was performed in 4 mL of pure CPME, the selectivity of furfural remained blocked at 2% due to the lack of the solubility of d-xylose in this organic solvent. These results confirmed the importance of a minimum of 1 mL of water as the reaction solvent. At 170 °C, upon irradiation in a closed system, water remained polar enough compared to CPME, and d-xylose is easily in contact with HCl. Overall, water is here the lower phase, and 1 mL seems to be enough despite the difficulty to control the inner heating temperature of the vessel aggravated by the great number of ions present in solution due to the dissociation of NaCl [29].

### 2.5. Influence of the Ratio Nafion NR50-NaCl on the Furfural Yield

**Figure 4:**
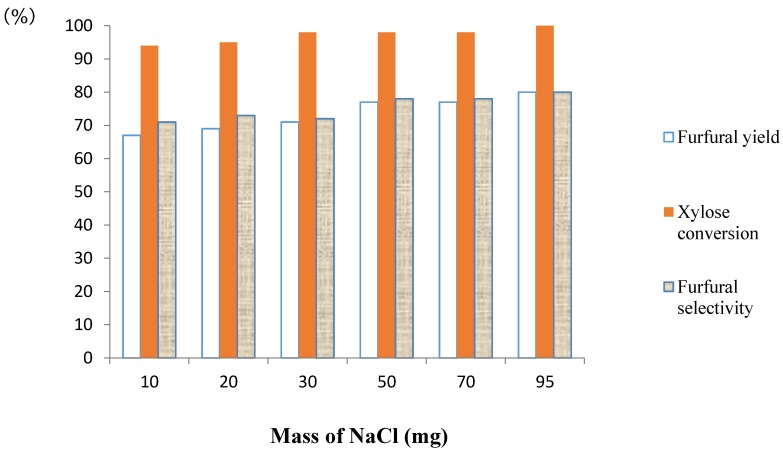
Effect of the amount of NaCl on the dehydration of d-xylose to furfural. Reaction conditions: Nafion NR50 (2 pellets), NaCl, d-xylose (1.0 mmol), water (1 mL), CPME (3 mL), MW, 170 °C and 40 min.

To the best of our knowledge, the inner structure of the Nafion NR50 pellets remains unclear, but all sulfonic acid functions were supposed to be accessible. In our process, when NaCl was added to the aqueous phase, a cation exchange between the Na^+^ of the salt and the H^+^ of the sulfonic acid groups of Nafion occurred, and HCl was released in the aqueous phase. Besides, the salt formed a coating on the pellets and seemed to protect them from the deposition of humins and, thus, from explosion. The impact of the ratio of Nafion NR50-NaCl on the dehydration of d-xylose process was investigated using the optimized method described above: Nafion NR50 (two pellets), NaCl, d-xylose (1.0 mmol), water (1 mL), CPME (3 mL), MW, 170 °C and 40 min. As reported in Figure 4, the mass of NaCl was progressively increased until a maximum of selectivity was obtained with 95 mg of NaCl. However, with only 50 mg of NaCl, the reactions started to furnish better selectivities (78%). This result corroborates a recent study that demonstrates the impact of increasing the concentration of NaCl on the produced furfural yield [30]. The same protocol was realized using only one pellet of Nafion NR50 (45 mg), but furfural was obtained in a lower yield (72%) (Figure S2, ESI).

To complete our study, NaCl was replaced by other inorganic salts, such as KCl or LiCl. In relation to the cation size and their relative mobility inside the Nafion pellets, a competition between water and the positively-charged species was expected to have an influence on the release of the protons in the reaction. Nevertheless, by using the same salt molar ratio, no variation of pH was recorded, and the yield of produced furfural was constant with a value of 80%.

Nafion NR50 has a capacity of 0.8 mmol of H^+^ per gram. For two pellets and an excess of NaCl, it corresponded to a pH of 1.1 in the vial if the totality of the protons was released in the medium. The pH of our reaction media reached a value of 1.3 on average, which means that a part of the sulfonic groups grafted on the resin remained protonated. To verify if the d-xylose conversion was only catalyzed by the HCl released in the medium or also by the resin, a reaction with an HCl solution at a pH of 1.3 (0.05 mol·L^−1^) was performed for 40 min in the presence of 90 mg of NaCl at 170 °C in water-CPME,1:3, *v*/*v*. A lower furfural yield of 73% was obtained instead of the 80% reached with the mixture of Nafion and NaCl. All of these observations permitted establishing that a new acid catalytic system was obtained having a synergetic effect between Nafion NR50 and NaCl. 

### 2.6. Reusability of the Nafion NR50

**Figure 5:**
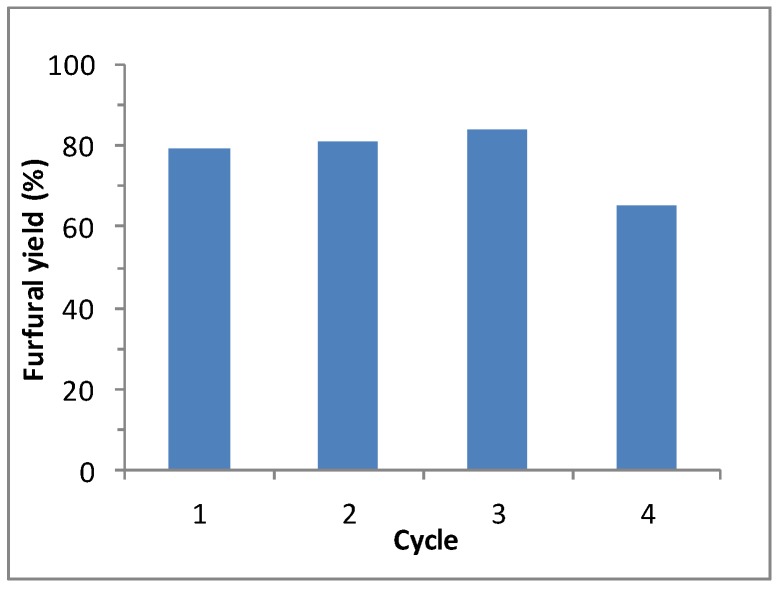
Reusability of the aqueous phase containing Nafion NR50 and NaCl for the d-xylose dehydration to furfural. Reaction conditions: Nafion NR50 (2 pellets), NaCl (95 mg), d-xylose (1.0 mmol), water (1 mL), CPME (3 mL), MW, 170 °C and 40 min.

Several consecutive reactions were performed with the same Nafion NR50 pellets. After each reaction, the resin pellets were recovered from the reaction medium and regenerated in an acidic HCl bath overnight. The reactions were performed for 40 min at 170 °C in water-CPME, 1:3, *v*/*v*. The activity of the Nafion NR50 pellets was kept constant for three consecutive cycles (Figure 5). After the fourth cycle, the furfural yield began to decrease. This observation can be explained by the gradual deactivation of the pellets by humin deposition.

### 2.7. SEM Observations EDX and Analysis of Nafion NR50 Pellets

**Figure 6:**
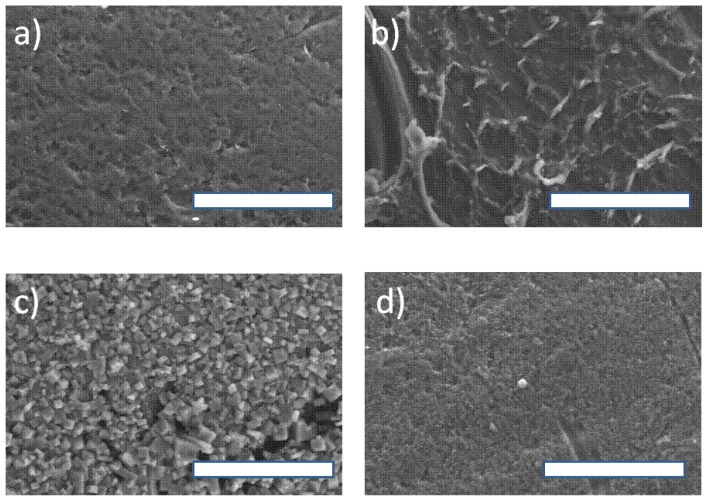
FE-SEM images of the Nafion NR50 surface morphology obtained from: (**a**) pristine Nafion; (**b**) the reaction without salt; (**c**) with salt after one catalytic run; (**d**) after regeneration in concentrated HCl solution (scale bar = 50 µm).

For each stage of our study, the morphology of the Nafion resins was observed by SEM. The photographs of these observations are provided (Figure 6) in association with a series of EDX analysis spectra (Figure S3, ESI). Figure 6a shows the fine porosity of the native Nafion NR50 surface. The EDX spectrum S3a gave the main constituents of Nafion: fluorine, carbon, oxygen and sulfur. The pellet analyzed in Figure 6b was used in a reaction without NaCl. The surface of the degraded catalyst was characterized by a network of cracks of various sizes and by the deposition of an organic residue on the surface. For this sample, the EDX spectrum S3b showed an enrichment of the catalyst with carbon and oxygen, evidence of the humin deposition. When the reaction was conducted in a NaCl aqueous solution, the recovered Nafion NR50 pellets (Figure 6c) appeared to remain intact. However, the surface of the catalyst was entirely coated by NaCl crystals, an observation confirmed by the EDX analysis S3c. When these pellets were used without treatment in an aqueous HCl solution, a further catalytic run in the presence of d-xylose gave only furfural in a 21% yield. Indeed, because of the partial exchange of protons H^+^ with Na^+^ cations, the catalyst was partially deactivated. Finally, the Nafion pellet can be regenerated in an acidic bath of HCl to recover its activity. As we can see in the image (Figure 6d), the inorganic salt coating was completely removed by this treatment, and it was proven by the suppression of both the Na and Cl peaks on the corresponding EDX spectrum S3d.

### 2.8. Furfural Production from Xylan

**Figure 7:**
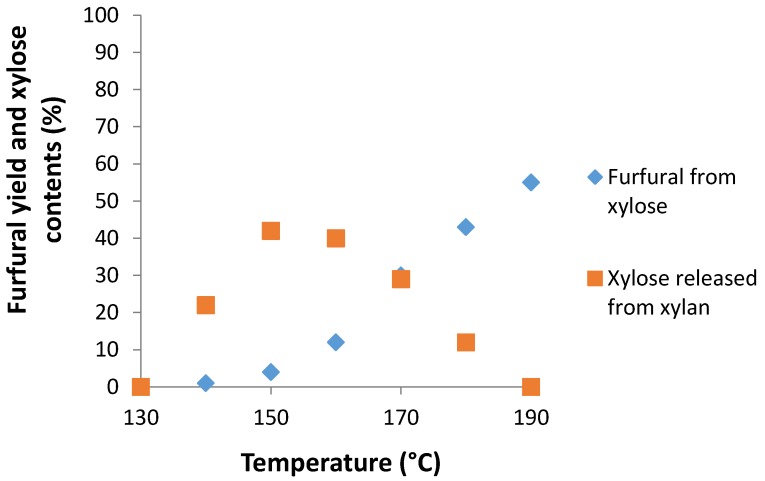
Furfural yield from xylan as a function of temperature for 60 min. Reaction conditions: Nafion NR50 (2 pellets), NaCl (95 mg), xylan (1.0 mmol), water (1 mL), CPME (3 mL), MW, 170 °C and 60 min.

The production of furfural from xylan involves a two-step reaction: a pseudo-first order irreversible depolymerization and a pseudo-first order dehydration. In our conditions optimized for the dehydration of d-xylose (Nafion NR50 (2 pellets), NaCl (95 mg), water (1 mL), CPME (3 mL), MW, 170 °C and 40 min), the xylan oligomer furnished the target furfural in a 19% yield. Thus, an increase of the time period (60 min vs. 40 min) and the variation of the temperature (130, 140, 150, 160, 170, 180 and 190 °C) were realized (Figure 7). As temperature rose from 130 °C to 160 °C, hydrolysis of xylan into d-xylose was accelerated, and the amount of sugar released in the media increased to reach a maximum of 42%. Then, from 140 °C, d-xylose was dehydrated into furfural, in a range of temperatures from this temperature to 170 °C, but the furfural concentration remained under the concentration of the released xylose. The yield increased from 30% to 55% with an increasing temperature from 170 °C to 190 °C. In regards to our results, the optimized conditions of furfural formation from xylan were identified: Nafion NR50 (2 pellets), NaCl (95 mg), water (1 mL), CPME (3 mL), MW, 190 °C and 60 min.

## 3. Experimental Section

### 3.1. General Information

Substrates were purchased from Acros Organic (d-xylose ≥ 99%, l-arabinose ≥99%), xylan from beech wood ≥90% from Sigma Aldrich (Saint-Quentin Fallavier, France). Catalyst (Nafion NR50) was purchased from Sigma Aldrich. Solvents were purchased from Acros (cyclopentyl methyl ether, Illkirch, France) and Fisher Scientific (acetonitrile, Illkirch, France). The standard (furfural 99%) was obtained from Acros. All materials were used without further purification. The water used in all experiments was Millipore Milli-Q grade. 

Each sample was analyzed separately by means of a Shimadzu Prominence HPLC. Pentoses were detected with a low temperature evaporative light scattering detector (ELSD-LTII, Shimadzu, Marne_la-Vallée, France), and furfural was detected with a UV-VIS detector (SPD-M20A, Shimadzu, Marne-la-Vallée, France) at a wavelength of 275 nm. The column used was a Grace Prevail C18 column (250 × 4.6 mm, 5 µm). The mobile phase was a MeOH–H_2_O (9:1) solution flowing at a rate of 0.5 mL·min^−1^. The column oven was set at 40 °C. d-Xylose conversion (*X*), furfural yield (*Y*) and furfural selectivity (*S*) were calculated by the following equations:

$$X=\frac{\left(Initial\;xylose\;amount\;\left(mol\right)-Final\;xylose\;amount\;\left(mol\right)\right)}{Initial\;xylose\;amount\;\left(mol\right)}\;\times 100$$

$$Y=\frac{Final\;furfural\;amount\;\left(mol\right)}{Initial\;xylose\;amount\;\left(mol\right)}\;\times 100$$

$$S=\frac{Furfural\;yield}{Xylose\;conversion\;}\times 100$$

Regularly, the calibration curve was checked in order to avoid eventual experimental errors associated with all measurements reported above. The results reported in all figures are averaged values calculated from three measurements.

SEM (scanning electron microscopy)-EDX (energy dispersive X-ray diffraction) analysis of Nafion pellets at different stages of the experiments was performed on a Quanta FEG 250 (FEI) equipped with a microanalysis detector for EDX (Brucker, Wissembourg, France). SEM micrographs acquired in secondary electron mode were obtained at low vacuum, 15 kV of accelerating voltage with a 10-mm working distance. EDX spectra were collected at a 30° angle, a 15-kV accelerating voltage and a 10-mm working distance.

### 3.2. General Procedure for the Synthesis of Furfural in a Water-CPME Biphasic Media

In a typical experiment, a 10-mL glass vessel was charged with water (1 mL), CPME (3 mL), the substrate (1.0 mmol), Nafion NR50 (2 pellets, ≈95 mg) and NaCl (1.62 mmol). The vessel was sealed with a septum, placed in the microwave apparatus (AntonPaar Monowave 300) and gradually heated for 5 min to the desired temperature via a resonant single mode under magnetic stirring (600 rpm) for the desired time. Temperature in the vessel was monitored by means of an IR sensor. At the end of the reaction, the vessel was cooled down to 40 °C using compressed air. Then, the two phases were separated. The aqueous phase was diluted in 200 mL of distilled water and filtered prior to analysis through a filter paper (10–20 µm, VWR, Fontenay-sous-Bois, France). The organic phase was diluted in 200 mL of acetonitrile and filtered prior to analysis through a syringe filter (PTFE, 0.45 µm, VWR). All experiments were repeated at least three times, and the deviation was lower than 5%.

## 4. Conclusions

In summary, this work reports a new efficient process for the production of furfural in an 80% yield. The method used a mixture of Nafion NR50 and NaCl in a water-CPME biphasic system at 170 °C under microwave irradiation. The association of Nafion NR50 and NaCl generated an unusual stability of the resin at 170 °C and a synergetic effect in acid catalysis. Regarding these promising results, the transposition of this methodology to cellulose or starch in order to produce 5-hydroxymethylfurfural (HMF) will be developed in due course.

## Supplementary Materials

The Supplementary Materials are available online at: http://www.mdpi.com/1420-3049/21/8/1102/s1.

## Abbreviations

The following abbreviations are used in this manuscript:

CPME	cyclopentyl methyl ether
DMSO	dimethylsulfoxide
EDX	energy dispersive X-ray
ELSD	evaporative light scattering detector
HPLC	high performance liquid chromatography
IR	infrared
MeTHF	methyltetrahydrofuran
MIBK	methyl isobutyl ketone
MW	microwave
PFA	polyfurfuryl alcohol
PTFE	polytetrafluoroethylene
rpm	revolutions per minute
SEM	scanning electron microscopy
THF	tetrahydrofuran
