# Supplementary material for: Furfural Production from d-Xylose and Xylan by Using Stable Nafion NR50 and NaCl in a Microwave-Assisted Biphasic Reaction
Source: Molecules. 2016 Aug 22;21(8):1102. doi: 10.3390/molecules21081102 (PMC6273969; doi:10.3390/molecules21081102)
Supplement: Supplementary file 1 [file molecules-21-01102-s001.pdf]

Supplementary Materials: Furfural Production from D-Xylose and Xylan by Using Stable Nafion NR50 and NaCl in a Microwave-Assisted Biphasic Reaction

Sarah Le Guenic, David Gergela, Claire Ceballos, Frederic Delbecq and Christophe Len

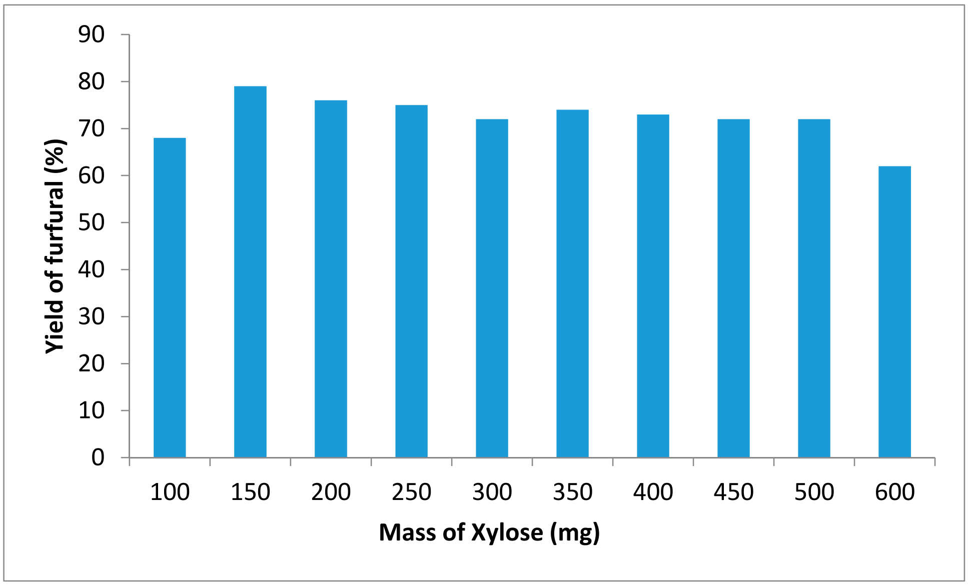

Figure S1. Effect of the initial xylose loading on the furfural yield. Reaction conditions: 95 mg of resins, 95 mg of NaCl, 1 mL of water and 3 mL of CPME heated in microwave oven at 170 °C for 60 min.

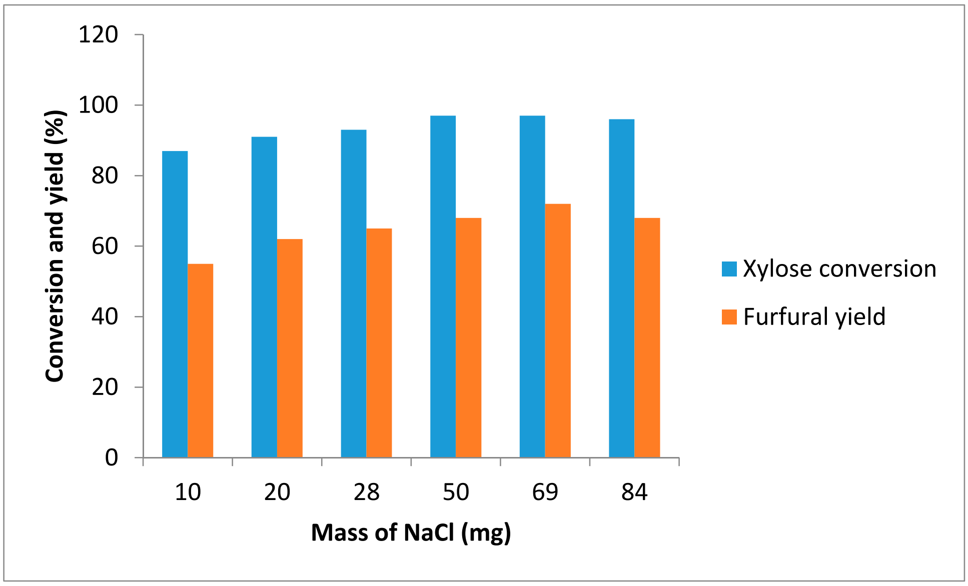

Figure S2. Conversion of the xylose and furfural yield versus the mass of NaCl employed; current condition: 150 mg of xylose (1.0 mmol), 1 Nafion pellet (45 mg), 1 mL of water and 3 mL of CPME, stirred at 170 °C for 60 min under a microwave oven.

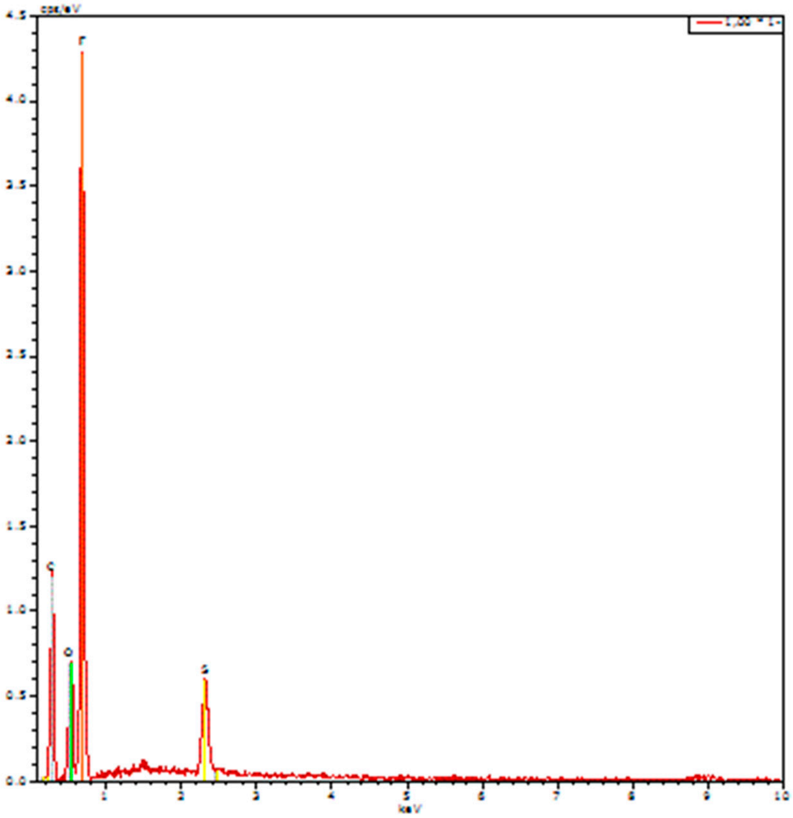

(**a**)

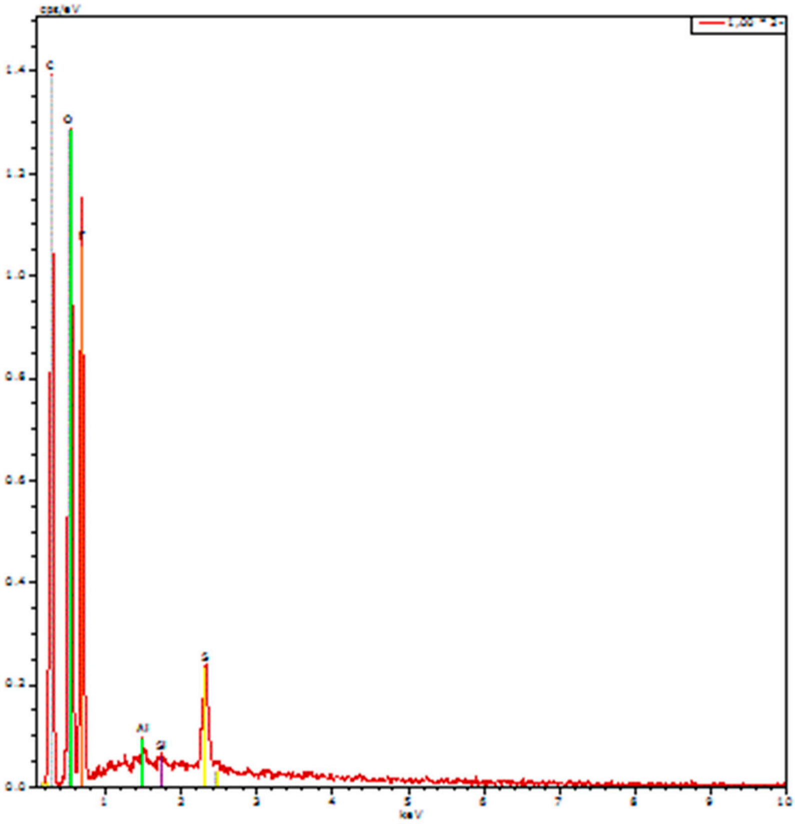

(**b**)

Figure S3. *Cont.*

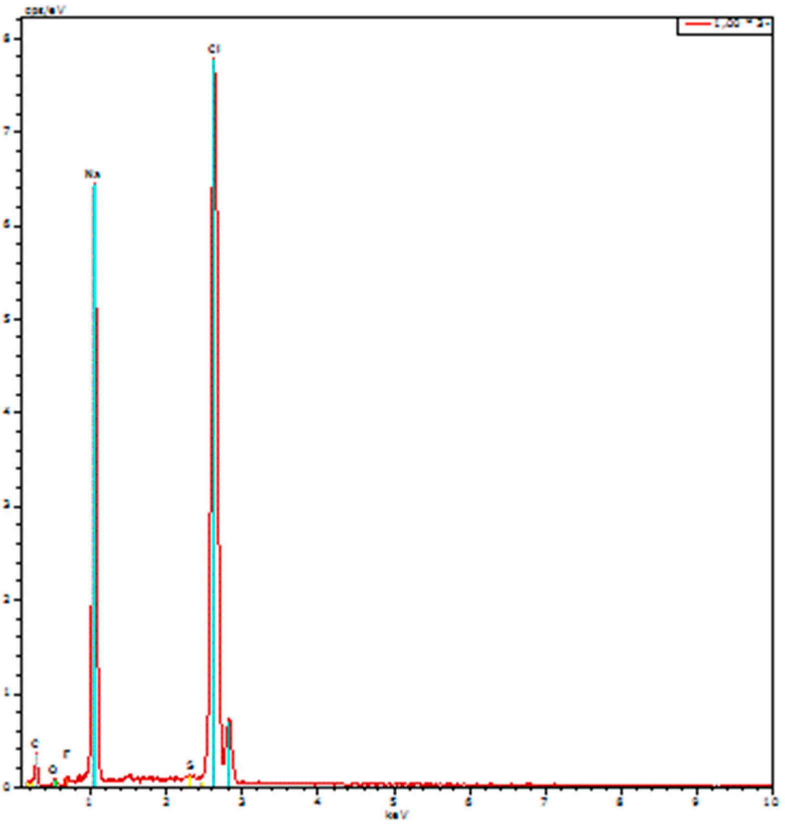

(**c**)

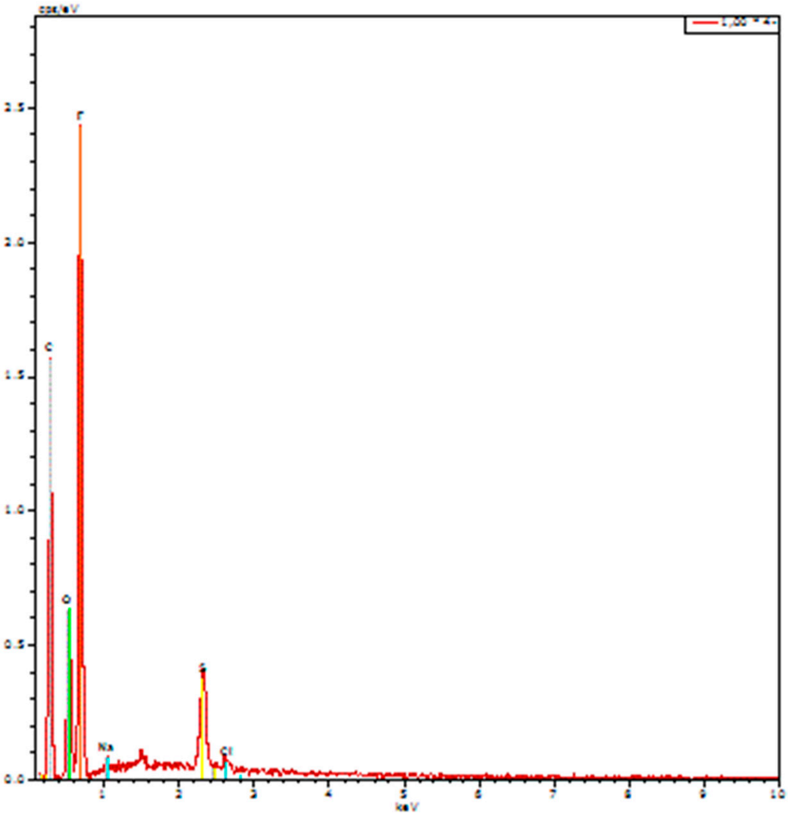

(**d**)

Figure S3. Energy dispersive X-ray diffraction (EDX) spectra of Nafion NR50 pellets at different stages of the process: (**a**) native material; (**b**) the reaction without salt; (**c**) after the reaction in the presence of NaCl; (**d**) after HCl bath immersion.
